# Deciphering Membrane Protein Complexes in *Plasmodium falciparum* Gametocytes via Integrative Structural Systems Biology

**DOI:** 10.1016/j.mcpro.2026.101567

**Published:** 2026-04-09

**Authors:** Siavash Mostafavi, Valentin Maurer, Max Ruwolt, Jennifer Schwartz, Frank Stein, Korbinian Niedermüller, Tim Wolf Gilberger, Fan Liu, Carolin Seuring, Susanne Witt, Jan Kosinski, Christian Löw, Michael Filarsky

**Affiliations:** 1 European Molecular Biology Laboratory Hamburg, Hamburg, Germany; 2 Centre for Structural Systems Biology, Hamburg, Germany; 3 Institute of Molecular Biology and Biophysics, ETH Zurich, Zürich, Switzerland; 4 Department of Structural Biology, Leibniz-Forschungsinstitut für Molekulare Pharmakologie (FMP), Berlin, Germany; 5 Proteomics Core Facility, European Molecular Biology Laboratory, Heidelberg, Germany; 6 Bernhard Nocht Institute for Tropical Medicine, Hamburg; 7 University of Hamburg, Hamburg, Germany; 8 Charité – Universitätsmedizin Berlin, Berlin, Germany; 9 Leibniz Institute of Virology, Hamburg, Germany; 10 University Medical Centre Hamburg Eppendorf (UKE), Hamburg, Germany; 11 Molecular Systems Biology Unit, European Molecular Biology Laboratory, Heidelberg, Germany; 12 Interfaculty Institute of Biochemistry, University of Tübingen, Tübingen, Germany

**Keywords:** *Plasmodium falciparum* gametocytes, cross-linking mass spectrometry (XL-MS), size-exclusion-based cofractionation mass spectrometry (CoFrac-MS), membrane proteomics, AlphaFold-modeling

## Abstract

Malaria is caused by protozoan parasites of the genus *Plasmodium* that proliferate asexually in human erythrocytes. During each replication cycle, a small fraction of the parasites differentiates into gametocytes. These sexual gametocytes are the only stages of the parasite that can infect the mosquito vector and transmit malaria. Their progression and maturation depend on a profound remodeling of the erythrocyte. This is achieved by the export of parasite proteins into the erythrocyte, leading to structural and mechanistic changes in the cytoskeleton and membrane of the host cell, crucial for gametocyte development. Since gametocytes are not susceptible to most antimalarials, they pose a major obstacle to current malaria intervention strategies. At the same time, they are a bottleneck within the parasite life cycle, making them an excellent target for future transmission-blocking interventions, which are critical in the context of malaria eradication efforts. Despite this, our current understanding of the composition and interactions within the gametocyte-specific proteome is limited, particularly with respect to membrane proteins and membrane-associated multiprotein complexes. To address this knowledge gap, we employed cross-linking mass spectrometry to detect residue-level proximities between proteins in the gametocyte membrane proteome. We then used a size-exclusion-based cofractionation mass spectrometry dataset as an orthogonal source of support for candidate protein associations. Furthermore, we modeled representative complexes using AlphaFold and AF3x, capable of using cross-links as restraints. By integrating these data types, we prioritized known and previously undescribed host–pathogen and pathogen–pathogen membrane-associated complexes distributed among the cellular compartments of the parasite and the erythrocyte host cell. The results of this work advance the molecular understanding of gametocyte biology and provide a valuable resource to inform future studies that have the potential to serve as a springboard for research aimed at transmission-blocking interventions.

## Highlights

• XL-MS and CoFrac-MS map membrane protein complexes in *P. falciparum* gametocytes.
• Novel membrane-associated parasite-parasite and host-parasite complexes identified.
• AlphaFold-based modeling predicts architectures of membrane protein complexes.
• Resource expands understanding of *P. falciparum* gametocyte membrane biology. - New interactions could potentially inform transmission-blocking interventions.

## Graphical Abstract

**Figure:**
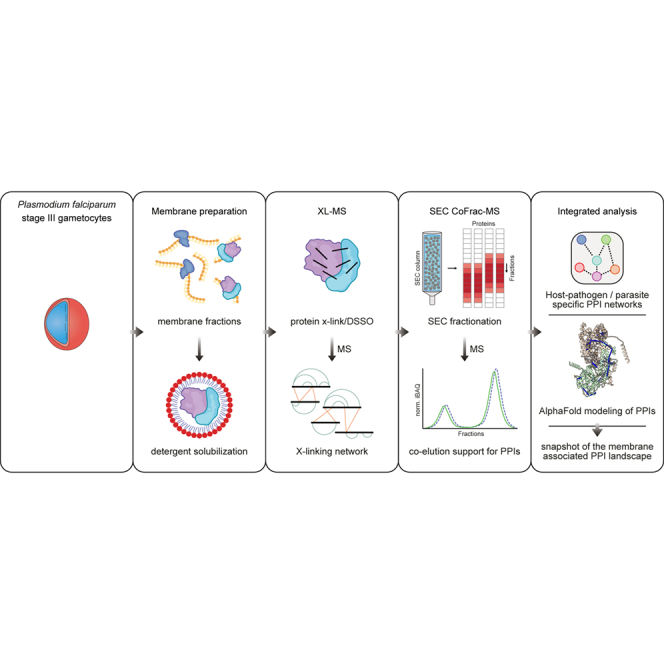

## In Brief

In early stage *Plasmodium falciparum* gametocytes, membrane proteins at the parasite-host interface drive gametocyte maturation and are essential for host erythrocyte remodeling. Despite this, the membrane protein complexes orchestrating these processes remain poorly defined. Using cross-linking mass spectrometry with orthogonal cofractionation experiments, this study maps interaction networks across parasite and host–cell membrane compartments. Integrating these data with AlphaFold-based complex modeling constrained by cross-links reveals structures for known and previously undescribed membrane-associated complexes. This resource advances our insights into essential gametocyte biology.

With an estimated 263 million cases and 597,000 deaths in 2023 alone, malaria remains a serious global health challenge (https://www.who.int/teams/global-malaria-programme/reports/world-malaria-report-2024). The majority of fatal cases of malaria can be attributed to the parasite *Plasmodium falciparum*, the most virulent human-infecting *Plasmodium* species (1). After an initial asymptomatic liver stage, the parasite progresses through iterative rounds of asexual replication within human erythrocytes, establishing a persistent infection of its human host (2). For successful transmission, the parasite relies on the differentiation of these intraerythrocytic asexual parasites into female and male gametocyte stages, which are essential for mosquito infection. Intraerythrocytic gametocyte development occurs in five morphologically discernible stages (I–V), over a time span of 10 to 12 days (3). Recent studies revealed that early-to-mid stage gametocytes (I–IV) sequester in host tissues such as the bone marrow and spleen and only enter back into circulation once they have developed into fully mature stage V gametocytes (4, 5, 6, 7). Since most current antimalarial drugs are less effective against gametocytes, they can persist in apparently cured infected individuals, causing ongoing transmission of the parasites in the community (8, 9). Gametocytes, therefore, present a major obstacle to malaria elimination efforts, and a better understanding of the underlying biology of gametocyte development, which helps the parasites evade drug pressure, will be crucial for successful malaria intervention strategies.

During their maturation process, gametocytes undergo profound cellular and molecular changes. This includes the formation of specialized membrane compartments, such as the inner membrane complex (IMC), the parasite plasma membrane (PPM), and the parasitophorous vacuole membrane (PVM), as well as the makeover of the cytoskeleton and the membrane of the host erythrocyte (10). It is mostly achieved in the early to midstages of gametocyte development (stages I-III), where about 10% of the proteins produced are exported out of the parasite to the parasitophorous vacuole (PV), the PVM, the erythrocyte cytosol, and the erythrocyte membrane (11). Many of these early exported proteins are gametocyte-specific and allow the parasite to remodel the erythrocyte cytoskeleton and surface properties, facilitating immune evasion and sequestration (12). Additional notable membrane-associated and integrated proteins include solute carrier transporters used by the parasites to mediate the uptake of essential nutrients and ions, ensuring metabolic homeostasis during the prolonged gametocyte development (13). Within the parasite, the IMC plays a crucial role in gametocyte biology. The IMC is a double membrane organelle, formed by 11 to 13 distinct plates connected through proteinaceous “sutures” underneath the plasma membrane (PM) (14). Current research suggests that the main function of the IMC is a structural role in driving the changes in size and shape observed during gametocyte development (14). Altogether, these membrane compartments and the remodeling of the host cell provide the basis for protein–protein interactions, which are critical for gametocyte differentiation, structural integrity, and host–parasite interactions. Despite their importance, the molecular composition and structural organization of membrane-associated protein complexes and their interactions with host proteins in gametocytes remain largely undefined. The limited research into multiprotein complexes in *Plasmodium* species has so far been centered on the readily available asexual stages of the parasite life cycle, and only very recent research has also included the gametocyte stages (15, 16, 17). In terms of structural information of potentially important membrane-associated complexes, there is even less data available, with a severe paucity of high-resolution protein structures from *P*. *falciparum* in comparison to other organisms (18).

To address this knowledge gap, we applied an integrative proteomic and structural modeling approach to elucidate the landscape of membrane-associated protein complexes of early-to-mid-stage gametocytes. We combined cross-linking mass spectrometry (XL-MS) and size-exclusion based cofractionation mass spectrometry (CoFrac-MS) of gametocyte cell extracts to capture and characterize putative membrane protein complexes in stage III gametocytes. XL-MS provided residue–residue proximity information within and between proteins, while CoFrac-MS offered orthogonal support of pairwise interactions through coelution profiles. By integrating these datasets with AlphaFold-based (19, 20) structural modeling, we were able to predict complex architectures and interaction interfaces. This combination allowed us to prioritize membrane protein complexes supported by complementary evidence from XL-MS, CoFrac-MS, and structural modeling, including traditionally hard-to-investigate membrane proteins.

Altogether, we present a proteomic snapshot of gametocyte membrane protein complexes, highlighting novel interactions that may contribute to essential processes such as vesicular trafficking, cytoskeletal remodeling, and membrane stability. Our study provides an overview of gametocyte membrane composition and host–pathogen interactions and illustrates how integrating proteomics with structural modeling and residue-level constraints can overcome limitations of previous approaches and expand structural understanding of *P*. *falciparum* biology.

## Experimental Procedures

### Experimental Design and Statistical Rationale

One batch of gametocytes was used for the CoFrac-MS. One batch of gametocytes was used as input material for the size-exclusion chromatography (SEC)-based cross-linking workflow and a mixture of three independent batches of gametocytes served as input for the strong cation exchange (SCX)-based cross-linking workflows. Nonfractionated peptides were analyzed by MSFragger to generate a target protein database, with protein-level false discovery rate (FDR) controlled at 1% to improve the robustness of the subsequent cross-link database searches by reducing the false-positive space. Ultimately, statistical confidence in the reported cross-link identifications is ensured through the FDR estimation implemented in the database search software used in this study, Scout, which is specifically designed to provide high-confidence cross-link identifications from large-scale interactomic studies. Separate intralink and interlink FDR was enabled and spectra and peptide-pairs were filtered to 2% and protein-protein interactions to 5% to balance sensitivity and specificity of a low complexity dataset. Due to the time- and resource-intensive nature of biomass production, cross-link enrichment, extended LC–MS acquisition, and downstream analysis, no technical or biological replicates were performed. Instead, two complementary enrichment strategies (SEC and SCX) were applied to samples from the same biological source to increase analytical depth and robustness. LC–MS system performance and reproducibility were monitored by repeated injections of a HeLa digest before and after the acquisition series and by bovine serum albumin injections every four fractions.

### Parasite Culture and Induction of Gametocytogenesis

*P*. *falciparum* NF54/iGP2 parasites containing the inducible GDV1-GFP-*glmS* expression cassette (21) were cultured in B+ human erythrocytes at 5% hematocrit in RPMI 1640 medium supplemented with 25 mM Hepes, 24 mM sodium bicarbonate, 100 mM hypoxanthine, and 0.5% AlbuMAX II (Life Technologies). In addition, 2.5 mM D-(+)-glucosamine hydrochloride (GlcN) was added to the cultures during routine parasite culture to maintain *glmS* ribozyme activity and inhibition of GDV1-GFP expression. Repeated sorbitol treatments were used for growth synchronization of asexual parasites (22). For the generation of gametocyte cultures, synchronous ring stage cultures at 1% parasitemia (8–16 hpi) in 5% hematocrit were washed in culture medium to remove GlcN and trigger sexual conversion by inducing expression of ectopic GDV1-GFP. After 48 h and one replication cycle, late ring stage cultures (16–24 hpi) were switched to culture medium containing 50 mM N-acetyl-glucosamine (GlcNAc) and the medium was changed daily until gametocytes reached stage III (day 5 after removal of GlcN) and asexual parasites were eliminated (23). To observe gametocytemia and assess gametocyte stages Giemsa-stained thin blood smears were visualized on a light microscope using a 63x immersion oil objective. For sample preparation stage III gametocytes were released from erythrocytes by saponin lysis (0.15% in PBS), followed by centrifugation at 4000 rpm for 10 min.

### Membrane Isolation, Solubilization, Fractionation and Cross-linking

Parasite pellets were resuspended in 20 mM Hepes pH 7.5, 150 mM NaCl, 5% glycerol, 5 μg/ml of RNAse A, 50U/ml DNase and 1x protease inhibitors. The cells were lysed by sonication (ON/OFF: 1s/29s, 10 min total time using TS 70 probe). The lysate was clarified by centrifugation at 15,000g for 15 min. The supernatant was then centrifuged at 100,000g for 1 h to recover the membranes. The membranes were then resuspended in the lysis buffer without DNase and centrifuged again at 100,000g. The membranes were then resuspended in 20 mM Hepes pH 7.5, 150 mM NaCl, 5% glycerol, 1x protease inhibitors. To solubilize the membranes, n-Dodecyl β-D-maltoside/cholesteryl hemisuccinate Tris salt (DDM/CHS) was added to a final concentration of 1%/0.1%, and the sample was incubated with mixing at 4 °C for 1 h before being centrifuged at 100,000g to remove insoluble material. Typical yields from a 300 ml batch of gametocytes yielded roughly 100-200 μg of total protein, depending on culture density.

To fractionate the sample, the clarified and solubilized material from ∼300 ml of culture was processed as described above. Then it was concentrated to 6.2 mg/ml (by *A*_280nm_) and injected onto a BioSep S-4000 size-exclusion column, at 0.3 ml/min in isocratic mode, in 20 mM Hepes pH 7.5, 150 mM NaCl, 0.03% DDM/0.003% CHS. In total, 61 fractions of 195 μL each, beginning slightly before the void volume and ending around an elution volume corresponding to a 44 kD gel filtration standard, were used for mass spectrometry (MS) analysis.

For cross-linking the sample, the same procedure for membrane isolation and solubilization was performed; however, the CHS used was not Tris-buffered. After solubilization, disuccinimidyl sulfoxide (DSSO) was added to a final concentration of 2 mM, and the sample was incubated at room temperature for 15 min. The reaction was quenched by the addition of 30 mM Tris–HCl pH 7.5. For the first dataset, material corresponding to 300 ml of gametocyte culture was used, while for the second dataset, 900 ml of cultured material was used.

### Sample Preparation, Data Collection and Analysis for the Size-Exclusion Chromatography Fractionated Sample

Protein samples were subjected to the SP3 protocol (24) conducted on the KingFisher Apex platform (Thermo Fisher Scientific). For digestion, trypsin was used in a 1:20 ratio (protease:protein) in 100 mM ammonium bicarbonate supplemented with 5 mM Tris(2-carboxyethyl)phosphine hydrochloride (TCEP) and 20 mM 2-chloroacetamide (CAA). Digestion was carried out for 5 h at 37 °C. Peptides were dried and taken up in 4% (v/v) acetonitrile (ACN) with 1% (v/v) formic acid (FA). An UltiMate 3000 RSLCnano LC system (Thermo Fisher Scientific) equipped with a trapping cartridge (μ-Precolumn C18 PepMap 100, 300 μm i.d. × 5 mm, 5 μm particle size, 100 Å pore size; Thermo Fisher Scientific) and an analytical column (nanoEase M/Z HSS T3, 75 μm i.d. × 250 mm, 1.8 μm particle size, 100 Å pore size; Waters). Samples were trapped at a constant flow rate of 30 μl/min using 0.05% trifluoroacetic acid (TFA) in water for 6 min. After switching in-line with the analytical column, which was pre-equilibrated with solvent A (3% dimethyl sulfoxide [DMSO], 0.1% FA in water), the peptides were eluted at a constant flow rate of 0.3 μl/min using a gradient of increasing solvent B concentration (3% DMSO, 0.1% FA in ACN). The gradient was as follows: 2% to 8% in 6 min (min), 8% to 25% in 39 min, 25% to 40% in 5 min, 40 to 85% in 0.1 min, maintained at 85% B for 3.9 min and re-equilibrated to 2% B for 6 min.

Peptides were introduced into an Orbitrap Fusion Lumos Tribrid mass spectrometer (Thermo Fisher Scientific) via a Pico-Tip emitter (360 μm OD × 20 μm ID; 10 μm tip, CoAnn Technologies) using an applied spray voltage of 2.2 kV. The capillary temperature was maintained at 275 °C. Full MS scans were acquired in profile mode over an m/z range of 300–1,500, with a resolution of 120,000 at m/z 200 in the Orbitrap. The maximum injection time was set to 250 ms, and the automatic gain control (AGC) target limit was set to 50%. The instrument was operated in data-dependent acquisition mode, with MS/MS scans acquired in the Ion trap in rapid scan mode. The maximum injection time was set to 35 ms, with an AGC set to “standard”. Fragmentation was performed using higher-energy collisional dissociation with a normalized collision energy of 30%, and MS2 spectra were acquired in centroid mode. The quadrupole isolation window was set to 1.6 m/z, and dynamic exclusion was enabled with a duration of 60 s. Only precursor ions with charge states 2 to 7 were selected for fragmentation.

Raw files were then searched using MaxQuant (version 2.3.0.0) (25) against the FASTA databases UP000005640_HomoSapiens_ID9606_20594entries_26102022_dl11012023 and UP000001450_PlasmodiumFalciparum_isolate3D7_ID36329_5372entries_060302023_dl17052023. The following modifications were included into the search parameters: Carbamidomethylation on C as fixed modification; Oxidation (M) and Acetylation (protein N terminus) as variable modifications. For the full scan (MS1) a mass error tolerance of 20 PPM and for MS/MS (MS2) spectra of 0.5 Da was set. For protein digestion, “trypsin” was used as protease with an allowance of a maximum of two missed cleavages, requiring a minimum peptide length of seven amino acids. Match between runs was enabled, as well as IBAQ value calculation. The FDR on the peptide and protein level was set to 0.01.

For the proteomics data analysis, the raw output file of MaxQuant (ProteinGroups.txt file files) was processed using the R programming environment (https://www.scienceopen.com/document?vid=300a2dc0-3207-4383-818c-51eb0f49f561). Initial data processing included filtering out contaminants and reverse proteins. Only proteins quantified with at least two unique peptides (with Unique.peptides ≥2) were considered for further analysis. Subsequently, 1690 proteins passed the quality control filters. For visualizations, iBAQ values were normalized by the maximum iBAQ value across all fractions and missing values were replaced by 0.

### Sample Preparation, Data Collection and Analysis for the Cross-linked Samples

#### Protein Digestion

DSSO cross-linked membrane fractions were digested using the SP3 procedure (26). Briefly, 4% SDS were added to the sample together with 1 μg Benzonase and incubated for 30 min at room temperature before homogenizing the sample for 10 min in a Bioruptor (30 s, 30 s off). Proteins were reduced with 5 mM TCEP and alkylated with 40 mM CAA for 1 h at room temperature. Sera-Mag beads were added to the sample followed by 50% ACN. The supernatant was removed after a brief incubation. The beads were washed twice with ethanol (EtOH) and once with ACN before the addition of digestion buffer, which contained 50 mM triethylammonium bicarbonate (TEAB) pH 8.5, LysC and Trypsin (both in enzyme:protein ratio 1:50 w:w). After incubation for 16 h at 37 °C, beads were washed twice by adding an excess of ACN. Peptides were eluted with 5% DMSO and dried until further use.

#### Peptide Fractionation

Cross-linked peptides were enriched by offline fractionation using either SEC or SCX. For SEC, 30 μg peptides were loaded onto a Superdex 30 Increase 3.2/300 (GE Healthcare) and separated over 60 min using 30% ACN with 0.1% TFA at a flow rate of 0.05 ml/min. Fractions were collected in intervals of 1 min, and early eluting fractions were combined into eight samples and dried prior to MS acquisition. For SCX, 80 μg peptides were loaded onto a PolySULFOETHYL A column 100 x 2.1-mm; 3 μm; 300 Å (PolyLC INC) and separated using a 57 min method with an effective 37 min gradient. Peptides were dissolved in 20% ACN containing 0.05% FA and eluted with increasing concentrations of 20% ACN with 0.05% FA and 0.5 M NaCl (0 min: 2%, 2 min: 3%, 7 min: 8%, 14 min: 20%, 23 min: 40%, 28 min: 90%, 37 min: 0%) at a flow rate of 0.18 ml/min. Fractions were collected in intervals of 1 min. Selected fractions were combined into 15 samples, dried, acidified, and desalted using C8 StageTips.

### Liquid Chromatography and Mass Spectrometry

Peptides were resuspended in 1% ACN, 0.05% TFA and injected into a Thermo Fisher Scientific Vanquish Neo system. Separation was performed using a PepMap C-18 trap column (0.075 mm × 50 mm, 3 μm particle size, 100 Å pore size, Thermo Fisher Scientific), followed by an in-house packed C18 analytical column (Poroshell 120 EC-C18, 2.7 μm, Agilent Technologies). Peptides were separated at a flow rate of 250 nl/min using a 177-min gradient of increasing ACN concentration, and analyzed on an Orbitrap Exploris 480 mass spectrometer equipped with a FAIMS Pro device (Thermo Fisher Scientific), operated with Instrument Control Software version 4.2. For MS1 scans, data were acquired in the Orbitrap at a resolution of 120,000, with advanced peak determination enabled. The MS1 acquisition settings were as follows: data type profile; m/z range of 375 – 1400; standard AGC target; maximum injection time 50 ms. Precursors with charges of +4 to +8 were isolated within a 1.6 m/z window and dynamically excluded for 60 s. MS2 scans were performed in the Orbitrap at a resolution of 45,000 with the following parameters: data type centroid; automatic scan range; 200% AGC target; 100 ms maximum injection time; stepped collision energies 27 ± 6%. Data acquisition cycled between FAIMS CVs −50, −60, and −75 with a cycle time of 2 s per compensation voltage (CV).

### Database Search

RAW data were analyzed using Scout version 1.5.1 (27) with the following parameters: DSSO as the cross-linker reacting with lysine, serine, threonine, and tyrosine side chains; trypsin digestion allowing up to four missed cleavages; peptide mass range 500–6000 Da; peptide length 6 to 60 residues. Precursor mass tolerance was set to 10 ppm and fragment mass tolerance to 20 ppm. Oxidation of methionine and protein N-terminus acetylation were included as variable, carbamidomethylation as a fixed modification. The interactome data were searched against a joined database containing 1028*P*. *falciparum* and 270 human proteins identified in a standard single-shot proteomics run against the human and the *P*. *falciparum* isolate 3D7 proteome (both retrieved from UniProt as one protein per gene) using MSFragger in Fragpipe version 22.0 (28) with default settings. Interlinks and intralinks were filtered separately to 2% FDR on both, spectrum and peptide pair level. Cross-linked protein pairs were additionally derived by applying FDR control at the PPI level at 5% (Supplemental Table S1). Peptide pairs have been exported from Scout and converted to a xiNET (29) compatible format for visualization. Two residue-pair files from independent experiments were concatenated to generate the combined dataset shown in Supplemental Table S1 in Scout format (A, All cross-links). For visualization, peptide pairs were exported from Scout and converted into a xiNET-compatible format. For counting total unique intraprotein and interprotein cross-links, a unique list was generated by retaining only the highest-scoring cross-link for each residue–residue pair. For structural analyses, ambiguous cross-links were expanded. Ambiguous cross-links arise when the cross-link site cannot be assigned to a specific residue within a peptide, for example, because the fragmentation pattern does not localize the modification to a single position, or when a cross-linked peptide sequence maps to multiple proteins with identical sequence over the cross-linked region. The expansion procedure involved enumerating all residue–residue and protein–protein combinations implied by the alternative residue or protein assignments. Each possible combination was listed explicitly, and the expanded list was then filtered to retain only unique residue pairs. The resulting list was used for structural analyses. Both the unique and expanded lists are provided in Supplemental Table S1, as (B) Unique cross-links and (C) Expanded cross-links.

### Comparison to Publicly Available SEC-MS Data

To further corroborate the cross-linked protein pairs identified in our XL-MS dataset, we compared it with publicly available SEC-MS datasets from PRIDE (PXD009039, PXD050751) and CEDAR (CRX23). MaxQuant results for each were downloaded from the respective repositories. From PXD009039, we selected four samples that profiled *P*. *falciparum* gametocytes: CH_FALC_ONE_15, CH_FALC_POINT_14, CH_FALC_POINT_27 and CH_FALC_ZERO_26. From CRX23, we used all four *P*. *falciparum* gametocyte samples (GAM1-4). All samples from PXD050751, which profiled *P*. *falciparum* schizonts, were included in the analysis.

Data were downloaded into R, and where necessary, PlasmoDB identifiers were converted to UniProt accessions using the UniProt REST API end point*/uniprotkb/search*. For each cross-linked protein pair in our XL-MS dataset, we calculated the Spearman correlation coefficient between their elution profiles across the SEC-MS fractions. High correlation values indicate that cross-linked proteins coelute, providing independent support for their physical interaction. For putative protein complexes (represented as graphs of cross-linked pairs), we computed the median correlation value across all protein pairs within each complex.

### Structural Modeling

For structural modeling, we first extracted connected clusters from the XL-MS network. Each binary interaction within the clusters was modeled individually, but we also modeled the full clusters up to the largest size that could be processed without exceeding available GPU memory and excluding well-known complexes such as the proteasome. For selected pairs and clusters, we generated models that included multiple copies of one or more proteins to test whether higher-order oligomeric states improved cross-link satisfaction. This was applied when prior evidence or violated cross-links suggested oligomer formation. AlphaFold 2 models were generated using AlphaPulldown v2.0 with five recycles. We used the latest official AlphaFold 2.3.2 sequence databases downloaded in February 2024 and template databases released between November 2024 and July 2025, depending (30). AlphaFold 3 was run using the official AlphaFold 3 implementation with default settings, including the sequence database downloaded in November 2024 and the Protein Data Bank (PDB) database (31) from November 2021, retrieved via the AlphaFold 3 download script (20). AF3x (32) was executed with 20 seeds and default parameters otherwise, using version #dfb94a3 and the same databases as AlphaFold 3. When multiple cross-links were linked to a given residue, a single random cross-link was used, prioritizing interchain cross-links. Model quality was evaluated using ipTM (interface predicted Template Modeling), pTM (predicted Template Modeling score), pLDDT (predicted Local Distance Difference Test), and PAE (Predicted Alignment Error) scores provided by the respective tools, as well as cross-link satisfaction. Structural visualizations were created with UCSF ChimeraX (33). PAE plots with cross-link overlay have been generated using PAE Viewer (34).

## Results

### Mapping Gametocyte Membrane Protein Interactions Using Integrative Proteomics

In order to investigate the membrane proteome of early *P*. *falciparum* gametocytes, we used the modified NF54 parasite line (NF54-iGP2), which enables the generation of synchronous, high gametocytemia cultures through the inducible overexpression of the protein gametocyte development 1 (GDV1) (21). Seven days after the induction of sexual commitment through activation of GDV1, followed by N-acetylglucosamine treatment to clear the remaining asexual parasites, stage III gametocytes were collected for sample preparation (Supplemental Fig. S1).

**Fig. 1:**
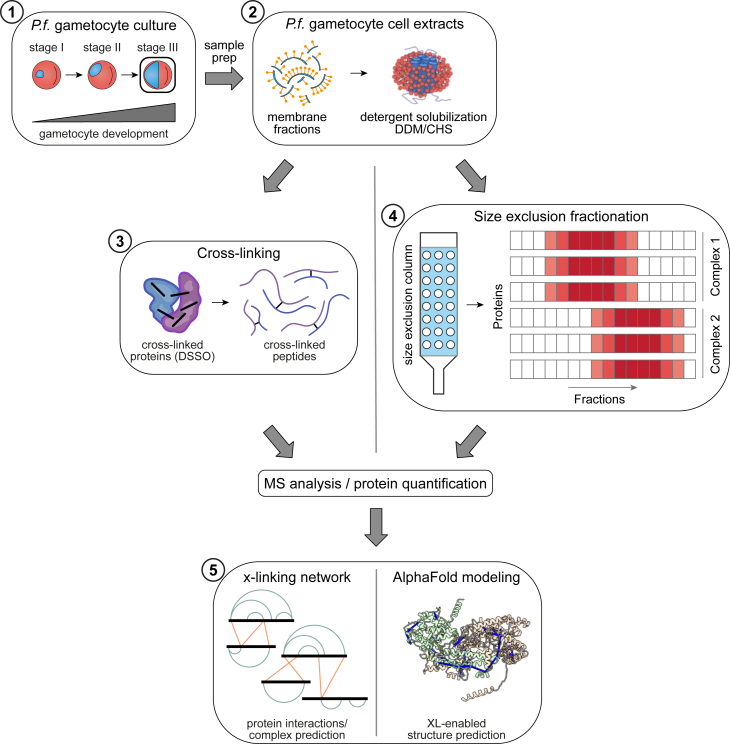
**Overview of experimental workflow**. Gametocytes were cultured from synchronized asexual parasite cultures through gametocytogenesis induced by GDV-1 overexpression (1). The gametocytes were harvested at stage III by saponin lysis of the iRBC. The isolated gametocytes were then lysed, and the membrane fraction was recovered by centrifugation, and solubilized in detergent (2). The solubilized material was then either cross-linked using DSSO (3) before MS analysis (XL-MS), or fractionated by size exclusion chromatography (4) before being analyzed by MS (CoFrac-MS). Identified complexes were modeled using AlphaFold (5). CoFrac-MS, cofractionation mass spectrometry; DSSO, disuccinimidyl sulfoxide; iRBC, infected RBC; MS, mass spectrophotometry; XL-MS, cross-linking mass spectrometry.

In summary, we generated three proteomics datasets. Two datasets after cross-linking, and one dataset after fractionation (Fig. 1). We collected the two cross-linking datasets using the gametocyte’s solubilized membrane fraction, followed by cross-linking with DSSO. The extent of cross-linking was assessed by SDS-PAGE (Supplemental Fig. S2). The first of these datasets was collected from a 300 ml batch of gametocytes at 5% hematocrit and ∼3% parasitemia at stage III, and the peptides were separated using SCX. The second dataset was collected from a 900 ml batch of gametocytes 5% hematocrit and ∼3 to 5% parasitemia at stage III, and the peptides were separated by SEC. For data analysis, the two datasets were combined. Interlinks and intralinks were filtered separately at 2% FDR at both spectrum and peptide-pair level. Protein-pair support at 5% PPI-level FDR was additionally derived from the same dataset and is reported to indicate which candidate protein interactions are also supported by more stringent XL-MS-based protein-pair filtering (Supplemental Table S1).

**Fig. 2:**
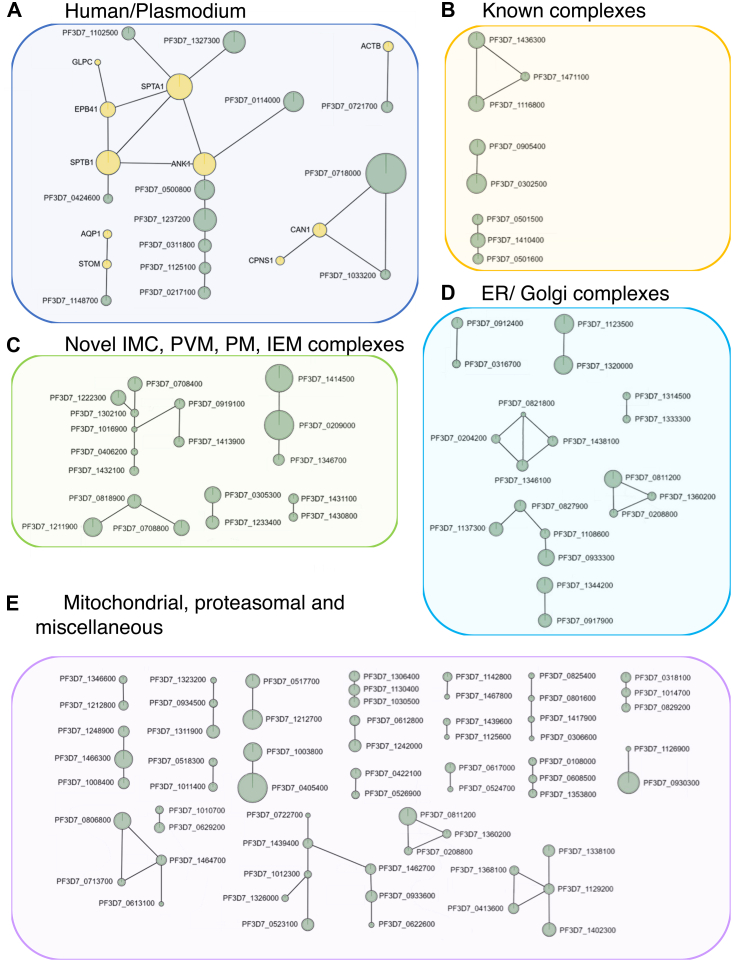
**The results of the XL-MS datasets visualized using xiNET** (29). The size of the circles represents the length of the protein, *green circles* represent parasite proteins, while *yellow circles* represent human proteins. *A*, cross-links between human and *Plasmodium falciparum* proteins. *B*, cross-links confirming previously described interactions within the IMC, PVM, IEM, and PEM compartments. *C*, novel cross-links between proteins found in the same compartments as *B*. *D*, cross-links between *P*. *falciparum* proteins found in the ER and Golgi. *E*, cross-links between mitochondrial, proteasomal, and miscellaneous proteins. ER, endoplasmic reticulum; IEM, infected erythrocyte membrane; IMC, inner membrane complex; PVM, parasitophorous vacuole membrane; XL-MS, cross-linking mass spectrometry.

In total, 258 interprotein cross-links and 1600 intraprotein cross-links were identified (Supplemental Table S1). The cross-linking network, excluding self-links and human-human cross-links, consists of 126 unique proteins, including 10 human proteins, spread across 39 disconnected subgraphs and is shown in Figure 2. In these, we recovered several known interactions between components of the *P*. *falciparum* respiratory chain complex, the proteasome and the core PTEX complex (Table 1), as well as previously described endogenous interactions within components of the human cytoskeletal network including Ankyrin-1 (ANK1, UniprotID: P16157), Spectrins (SPTA1, P02549, and SPTB, P11277), and Band 4.1 protein (EPB41, P11171), shown in Figure 2, box A. These serve as an internal validation of the datasets and support their reliability. We also mapped our cross-linking data onto previously published structures as an additional source of data validation (Supplemental Fig. S3).

**Table 1:** A list of miscellaneous (not directly mentioned in the text) and confirmatory complexes according to the XL-MS data

Name	Components	Category/compartment
ER membrane protein complex subunits	PF3D7_0811200, PF3D7_1360200, PF3D7_0208800	ER
PTEX core complex	PF3D7_1436300, PF3D7_1116800, PF3D7_1471100	PVM
26S proteasome regulatory subunits	PF3D7_1338100, PF3D7_1129200, PF3D7_1402300, PF3D7_0413600, PF3D7_1368100	Proteasome
26S proteasome regulatory subunits	PF3D7_1008400, PF3D7_1466300, PF3D7_1248900	Proteasome
Proteasome catalytic subunits	PF3D7_1011400, PF3D7_0518300	Proteasome
Proteasome catalytic subunits	PF3D7_0108000, PF3D7_0608500, PF3D7_1353800	Proteasome
-	PF3D7_1464700, PF3D7_0613100, PF3D7_0713700, PF3D7_0806800	Mitochondrion, general
-	PF3D7_1323200, PF3D7_0934500, PF3D7_1311900	Mitochondrion, general
-	PF3D7_1142800, PF3D7_1467800	Mitochondrion, general
-	PF3D7_0801600, PF3D7_0825400, PF3D7_1417900, PF3D7_0306600	Mitochondrion, general
-	PF3D7_0722700, PF3D7_1439400, PF3D7_1012300, PF3D7_1326000, PF3D7_0523100, PF3D7_1462700, PF3D7_0933600, PF3D7_0622600	Mitochondrion, respiratory chain
-	PF3D7_1125600, PF3D7_1439600	Mitochondrion, respiratory chain
-	PF3D7_0422100, PF3D7_0526900	Mitochondrion, respiratory chain
-	PF3D7_1242000, PF3D7_0612800	Unknown
-	PF3D7_1126900, PF3D7_0930300	Unknown
-	PF3D7_1212700, PF3D7_0517700	Unknown
-	PF3D7_0405400, PF3D7_1003800	Unknown

ER, endoplasmic reticulum; PVM, parasitophorous vacuole membrane; PTEX, *P*. *falciparum* translocon of exported proteins; XL-MS, cross-linking mass spectrometry.

**Fig. 3:**
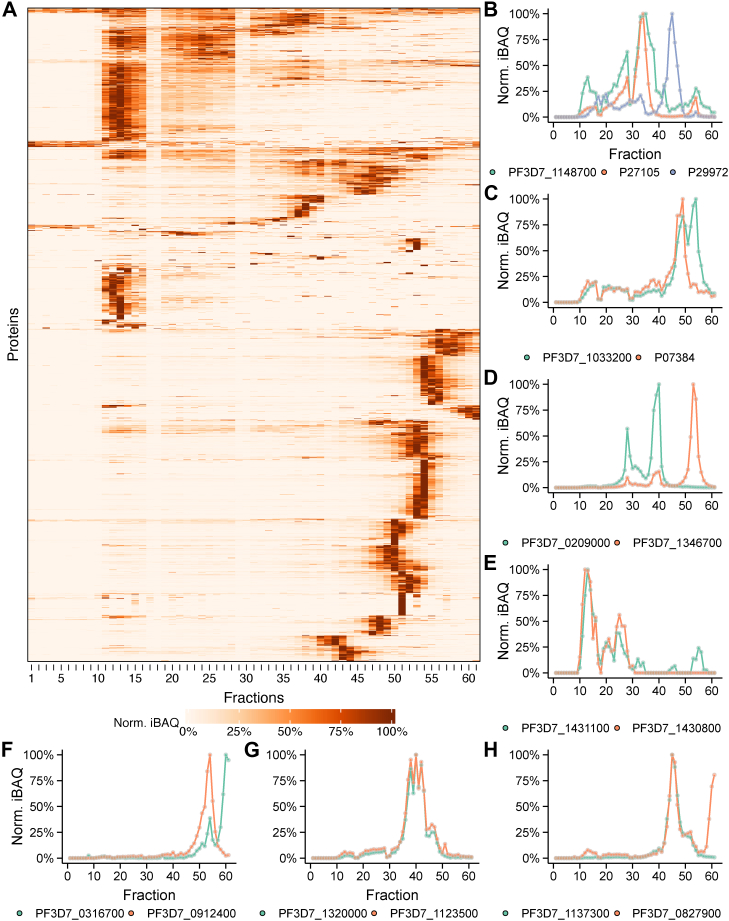
**Overview of the CoFrac-MS data**. *A*, heatmap of normalized iBAQ intensities (0–1) across the SEC fractions for all identified proteins. Each protein profile was normalized to its maximum iBAQ value and clustered using agglomerative clustering with complete linkage based on Euclidean distance. Missing iBAQ values were set to zero. *B*–*H*, individual elution profiles corresponding to specific complexes. The *Y*-axis represents the normalized iBAQ values, while the *X*-axis is the fractions. PlasmoDB IDs (or Uniprot IDs for human proteins) are indicated in the chromatograms. CoFrac-MS, cofractionation mass spectrometry; MS, mass spectrometry; SEC, size-exclusion chromatography.

The third dataset was collected after solubilization of the membrane protein fraction of stage III gametocytes from a 300 ml culture. The proteins were then fractionated by SEC, identified by MS and quantified across all fractions (Fig. 3*A*, Supplemental Fig. S4). This dataset resulted in 1238 *Plasmodium* proteins and 452 human proteins (Supplemental Table S2). Based on the subcellular localization annotation from UniProt, 564 proteins were considered membrane proteins. The SEC elution profiles served as an orthogonal dataset to corroborate XL-MS-identified interactions, with the expectation that interacting proteins would show correlated elution behavior. Individual elution profiles for representative complexes discussed below are shown in Figure 3, *B*–*H*. We further validated our findings against publicly available cofractionation mass spectrometry (CF-MS) datasets from independent studies (15, 16, 17) (see Experimental procedures, Supplemental Fig. S1, Supplemental Table S5). Although differences in parasite stages, culture conditions, fractionation strategies, and data processing workflows preclude quantitative comparisons or formal thresholds, several XL-MS-derived complexes show recurrent co-elution trends across datasets (for example, complexes 3, 25, and 26 in Supplemental Fig. S5). This provides additional orthogonal, albeit qualitative, support for the robustness of the identified interactions.

**Fig. 4:**
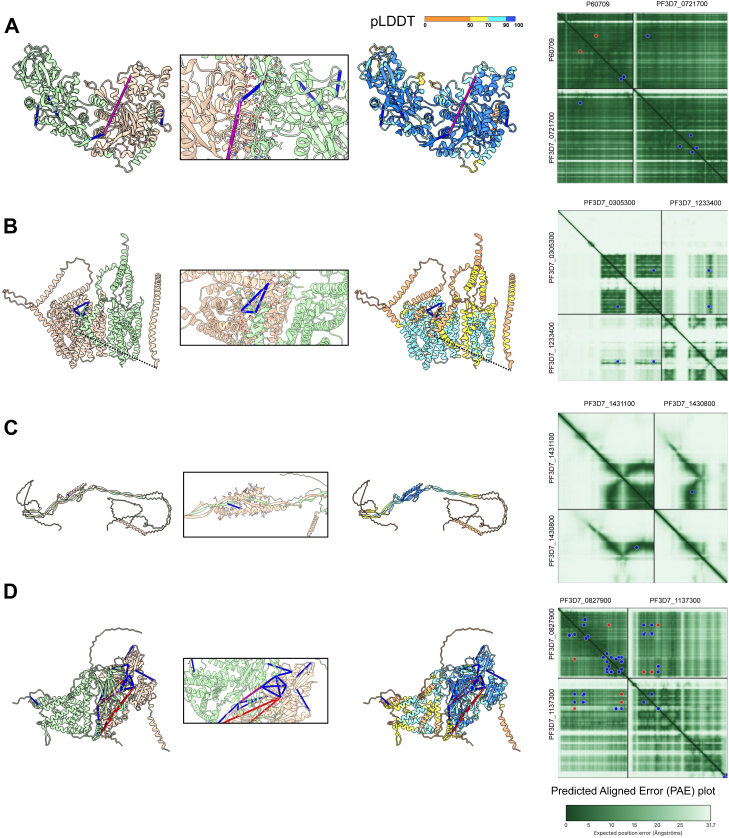
**Selected AlphaFold predictions of different complexes are shown**. Long, poorly scored, and ordered protein loops have been hidden from view for clarity. Cross-links are mapped onto the models and colored by Cα distance: *blue* (<30 Å), *purple* (30–40 Å), and *red* (>40 Å). *Left panel*: models colored by chain. *Middle left*: zoom-in on the predicted interaction interface. *Middle right*: models colored by pLDDT (predicted local distance difference test) confidence scores: *blue* (>90, very high), *light blue* (70–90, confident), *yellow* (50–70, low), and *orange*/*red* (<50, very low). *Right panel*: predicted aligned error (PAE) plot, showing the confidence in relative domain and chain positioning, with *dark green* indicating very high confidence. In addition, cross-link positions are shown as *blue* or *red dots* (based on PAE Viewer (34), depending on cross-link distance, with *red dots* indicating marginal or unsatisfied cross-links with a Cα distance over 30 Å. *A*, the human Actin–PSOP1 (P60709 and PF3D7_0721700) AlphaFold 3 model shows very high confidence and satisfies the single interprotein cross-link available and three out of four intraprotein cross-links. *B*, the PF3D7_0305300 and PF3D7_1233400 AlphaFold 3 model depicts a canonical MFS transporter in complex with a GPCR-like membrane protein and satisfies two out of two interprotein cross-links and the single intraprotein cross-link available. *C*, the PIP2–PIP3 (PF3D7_1431100 and PF3D7_1430800) AlphaFold 2 model displays an unusual elongated, twisted pair β-sheet arrangement, but is well validated by scores and satisfies the single interprotein cross-link available. *D*, the PF3D7_0827900 and PF3D7_1137300 AF3x model satisfies nine out of 12 interprotein cross-links and all 14 intraprotein cross-links. MFS, major facilitator superfamily.

Furthermore, we employed Alphafold 2 (19), AlphaFold 3 (20), and AF3x (32)—an extension of AlphaFold 3 that incorporates cross-link-derived restraints— to model the identified host–pathogen and pathogen–pathogen interactions, selecting pairs based on novelty and biological function (Supplemental Table S3). We included AlphaFold 2 in addition to AlphaFold 3 because AlphaFold 3 has more restrictive licensing, which can limit reuse of models in some settings, whereas AlphaFold 2 remains broadly usable. Moreover, the two methods rely on different network architectures, and agreement between AlphaFold 2 and AlphaFold 3 provides additional confidence, while AlphaFold 2 can succeed where AlphaFold 3 fails due to missing contextual factors or stochastic effects (35). Model reliability was supported by iPTM scores, PAE plots, and pLDDT scores, provided by AlphaFold, the concordance of cross-linking distance restraints, and agreement between the three methods. Whenever unconstrained AlphaFold 2 or AlphaFold 3 predictions yielded well-defined and high-confidence models, these were prioritized, and cross-links were used for validation rather than active restraints. AF3x improved cross-link satisfaction and model scores; however, in many cases, the additional satisfied restraints mapped to disordered regions or flexible loops and thus had limited impact on the predicted interface geometry or global architecture. Selected predictions from all three methods are included in Figure 4. The evaluations for all modeled complexes can be found in Supplemental Table S3 and Supplemental Fig. S6 (a link to an online repository containing the models is given in the Data Availability section).

In the integrated analysis, XL-MS provided residue-level proximity information and peptide-pair-level support for protein associations, with additional annotation of protein pairs supported at 5% PPI-level FDR. CoFrac-MS and AlphaFold-based structural modeling provided orthogonal evidence for prioritization and interpretation of candidate complexes. Supplemental Table S4 summarizes the supporting evidence for the protein pairs.

### Host–Pathogen Interactions

#### *P*. *falciparum* Interactions With the Red Blood Cell Cytoskeletal Network and the Human Ankyrin Complex

Due to the nature of saponin lysis, we reasoned that strongly interacting human proteins bound to *P*. *falciparum* proteins could also be identified across our datasets. Consistent with this expectation, human–Plasmodium cross-links were identified. The remodeling of the red blood cell (RBC) during gametocyte development is largely achieved through interactions of exported parasite proteins with the spectrin–actin network of the RBC cytoskeleton and the ankyrin complex linking the cytoskeletal network to integral erythrocyte membrane proteins (10, 12, 36). Accordingly, most of the host–pathogen cross-links we captured are related to cytoskeletal adhesion or remodeling (Fig. 2, box A). We could identify cross-links between the PHIST family proteins PF3D7_1102500 (GEXP02) and PF3D7_0424600 with spectrin alpha and beta (P02549, P11277) as part of a larger cross-linking network consisting of band 4.1 (EPB41, P11171), glycophorin-C (GLPC, P04921), and ankyrin-1 (ANK1, P16157). We furthermore observed cross-links between ankyrin-1 and the parasite proteins PF3D7_0114000 (GEXP06) and PF3D7_0500800 (MESA). Altogether, these results confirm previously described interactions of these proteins with the erythrocyte cytoskeletal network, generated by co-immunoprecipitation (co-IP) studies (37, 38) and further support our experimental approach.

We also identified cross-links of previously undescribed host–pathogen interactions. PF3D7_1148700, a gametocyte-enriched and putative host cytoskeletal interacting protein, is cross-linked to human stomatin (P27105). Stomatin is, in turn, cross-linked to human aquaporin-1 (P29972), an interaction that has been previously described within the context of the larger ankyrin complex (39, 40). Thus, PF3D7_1148700 may be an additional factor involved in host cytoskeletal remodeling through its interaction with the ankyrin complex. These three proteins also co-elute in the CoFrac-MS dataset (Fig. 3*B*).

Another intriguing cross-linking network was identified consisting of the two *P*. *falciparum* proteins early transcribed membrane protein 10.2 (ETRAMP 10.2, PF3D7_1033200) and the dynein heavy chain (PF3D7_0718000), which both cross-linked to human calpain-1 (P07384/CAN1). The putative interactions are partly supported by the CoFrac-MS dataset with ETRAMP 10.2 and calpain-1 co-eluting, while the dynein heavy chain is absent from the CoFrac-MS data (Fig. 3*C*). The molecular function of ETRAMP 10.2 in gametocytes is not established yet. It contains a predicted signal peptide, a transmembrane anchor, and a charged PHIST domain and is predicted to be exported to Maurer’s clefts and the host cytoskeleton (41, 42). Within this context, its potential interaction with calpain-1, a human protease involved in the regulation of cytoskeletal remodeling (43) and parasite egress in asexual parasites (44), could indicate that the interaction of these two proteins plays a similar role during the pronounced cytoskeletal remodeling required during gametocyte development or as a prerequisite for egress. In addition, we identified a cross-link between PSOP1 (PF3D7_0721700) and human Actin B (ACTB, P60709). PSOP1 has recently been found as part of the secreted proteome of activated gametocytes, suggesting a possible function in mediating the egress from the host cell (45, 46). Although there are no activated gametocytes in the sampled cultures, it is possible that the interaction was picked up because the cross-linking was performed after cell lysis and solubilization, bringing PSOP1 and human actin B in premature contact. In the CoFrac-MS dataset, the two proteins elute toward the end of the column (as would be expected from proteins of this size), close to each other; however, they do not perfectly overlap. Despite previous reports suggesting poor AlphaFold 2/3 performance within the context of modeling interspecies interactions (47, 48, 49, 50), modeling of PSOP1 (PF3D7_0721700) and Actin B (ACTB, P60709), yielded a model with high ipTM score of 0.89, interface residues pLDDT scores above 90, and PAE plots with the low expected error below 15 Å around the interaction interface (Fig. 4*A*). The modeled structure satisfied three out of four intraprotein cross-links with a 30 Å distance cutoff, as well as validating the interprotein cross-link, which has an apparent distance of ∼10 Å. The model was also consistent between AlphaFold versions 2 and 3 (Supplemental Table S3).

### Plasmodium Complexes

#### Infected Erythrocyte Membrane, PVM, PM and IMC

Within the parasite cell compartments, we recapitulated several previously described interactions (Fig. 2, box B). Among these were the three core components of the P. falciparum translocon of exported proteins (PTEX) complex (PF3D7_1436300, PF3D7_1471100, PF3D7_1116800). The PTEX translocon is located within the PVM where it is essential for exporting parasite proteins into the infected RBC (iRBC) setting the basis for the extensive remodeling of the iRBC by the parasite during gametocyte development (11, 51). We also recapitulated the interaction between RhopH3 (PF3D7_0905400) and cyto-adherence linked protein 3.1 (CLAG3.1) (PF3D7_0302500) (52). Together with RhopH2 (PF3D7_0929400) these proteins form the RhopH complex and have been shown to localize to the PVM and the iRBC membrane in asexual parasites (53). Within this context, the RhopH complex controls iRBC permeability by creating new permeability pathways for nutrient uptake (54, 55). The identification of the RhopH3/CLAG3.1 cross-linking pair in our samples underscores recently published findings that revealed the presence of RhopH2 in immature gametocytes by immunofluorescence and also showed new permeability pathway activity persists in early stage gametocytes up until they reach stage III, suggesting the continued presence and function of the RhopH complex in these developmental stages (13). In addition, we identified cross-links between rhoptry-associated protein 1 (RAP1, PF3D7_1410400) and rhoptry-associated proteins 2 and 3 (RAP2/3, PF3D7_0501600, PF3D7_0501500). Initially thought to be important during merozoite invasion into the RBC, the RAP complex has been shown to reside within the PVM after reinvasion during ring and trophozoite stages in *Plasmodium berghei*. The conditional knockdown of RAP2 resulted in defects in the PVM ultrastructure and a delayed growth and reduction in parasitemia, indicating a role of the RAP complex in organizing the PVM architecture in these stages (56). Affinity purification experiments of RAP1 in *P*. *falciparum* found several PVM-associated proteins copurifying with RAP1, including RhopH3, and the PTEX translocon, which matches the proteins identified in our samples (56). This could point to a similar role for the RAP complex in the organization of the PVM in immature gametocytes. We also captured the previously described interaction between transmission-blocking target Pfs230 (PF3D7_0209000) and Pfs45/48 (PF3D7_1346700) in both the XL-MS (Fig. 2, box C) and CoFrac-MS (Fig. 3*D*) datasets (57). Interestingly, Pfs230 is also cross-linked to an atypical putative protein kinase (PF3D7_1414500), although this kinase is absent from the CoFrac-MS dataset. Pfs230 is a critical, high-value therapeutic target, and is known to be phosphorylated on multiple sites, making an interaction with a kinase responsible for its function or regulation plausible.

In addition to known complexes, we identified several cross-links within a subnetwork indicating potentially novel protein interactions (Fig. 2, box C). Pfs16 (PF3D7_0406200), a gametocyte-specific PVM protein important for gametocyte development and transmission (58, 59, 60) cross-links with a voltage-dependent anion channel (PF3D7_1432100). PF3D7_1432100 was recently described as a porin protein localized to the PPM and putatively involved in the export of membrane proteins into the PVM (61). The protein was shown to be essential for asexual replication of the parasite and the observed cross-link between PF3D7_1432100 and Pfs16 indicates an interesting interaction of these two proteins at the parasite erythrocyte interface during gametocyte development. We also detected a cross-link of Pfs16 with the early transcribed membrane protein 10.3 (ETRAMP10.3, PF3D7_1016900), which has so far been described as a PVM localized protein in asexual and liver stages of the parasite (62). ETRAMP10.3 in turn cross-links with gamete antigen 27/25 (PF3D7_1302100), an abundant protein in early gametocytes involved in maintaining cell integrity during gametocyte maturation (63). The additional proteins in this cross-linking subcomplex are the chaperones PfHSP90 (PF3D7_0708400) as well as PF3D7_1222300 (endoplasmin, (GRP94)) which have been indicated as ETRAMP10.3 interaction partners previously (64). Although the localization of these three proteins is largely described as cytoplasmic and endoplasmic reticulum (ER)-associated (GRP94), a loss of Pfg27 resulted in a fraction of gametocytes, displaying distorted membrane structure, vesicles, and random vacuoles of largely PVM and ER-derived components (63). The observed cross-link between Pfg27 and the PVM protein ETRAMP10.3 underscores these findings and, in addition, is in line with recent observations of contact points between the ER and PPM in developing gametocytes (65).

Another cross-link network in Figure 2, box C involves the sodium homeostasis pump PfATP4 (PF3D7_1211900) and the two heat shock proteins PfHSP70 (PF3D7_0818900) and PfHSP110 (PF3D7_0708800). PfATP4 is an essential sodium efflux pump in the PPM and has been characterized as a promising drug target, in asexual parasites and gametocytes alike (66, 67, 68). Similarly, PfHSP70 and PfHSP110 have been shown as viable drug candidates. Although an interaction between PfHSP70 and PfHSP110 is well established, a connection to PfATP4 has so far not been shown. The observation of a putative interaction of PfATP4 with the PfHSP70/110 complex could indicate a functional relationship between these proteins or a dependency of PfATP4 on PfHSP70/100 for proper folding or stability that could be exploited in the context of future intervention strategies.

The putative major facilitator superfamily transporter PF3D7_0305300 was cross-linked to the conserved *Plasmodium* membrane protein PF3D7_1233400 (Fig. 2, box C), both of which were previously identified as immunogenic and have been individually explored as potential vaccine targets (69, 70). PF3D7_0305300 is abundantly expressed in gametocytes, where it localizes to the gametocyte PM or PVM, potentially playing a role in metabolite transport between the gametocyte and the iRBC (69). PF3D7_1233400 is a predicted membrane protein that appears to be expressed specifically in gametocytes (11, 71). Antibodies against the protein can be readily detected in natural infections and correlate with a reduced parasite burden, suggesting the accessibility of the protein for the immune system and again pointing to a membrane or gametocyte surface localization of the protein (70). Besides this, the biological function of PF3D7_1233400 is undescribed. The AlphaFold model of this complex satisfied all three cross-links, including the interprotein cross-link, revealing what appears to be a canonical major facilitator superfamily transporter interacting with a protein that contains at least six apparent transmembrane helices (Fig. 4*B*). Although the model exhibited rather low ipTM score of 0.43, the models from all AlphaFold versions agreed with each other and had good local pLDDT and PAE scores around the interaction interface. Taken together, the obtained data indicate that the two proteins form a novel complex involved in gametocyte metabolite transport, which would be a prime candidate for further study given their indicated potential as an intervention target.

Within the IMC, a double-membrane structure underneath the PPM with a complex protein composition, we found cross-links between Photosensitized INA-Labelled protein 1 (PhIL1) interacting proteins PIP2 (PF3D7_1431100) and PIP3 (PF3D7_1430800) (Fig. 2, box C), which also co-elute in the CoFrac-MS dataset (Fig. 3*E*). PhIL1 was shown to be essential for the maturation of gametocytes (72, 73); however, it is absent from the XL-MS and CoFrac-MS datasets, thus it is possible that PIP2 and PIP3 independently form a complex without PhiL1. The AlphaFold model of this complex showed an elongated structure with unusual, long stretches of twist-pair wrapping between beta sheets (Fig. 4*C*). The ipTM score of the model is low, but the models from all three AlphaFold versions were similar and exhibited good pLDDT and PAE scores around the interface and satisfied the interprotein cross-link.

#### Endoplasmic Reticulum/Golgi

Immature gametocytes harbor an extensive ER, which occupies large parts of the cell (65) and functions as a protein processing and transportation hub for parasite proteins that are exported to the erythrocyte, thereby playing a role in the remodeling of the host cell (74). Within the ER (Fig. 2, box D), we identified cross-links between YOP1 (PF3D7_0316700) and an alkaline phosphatase (PF3D7_0912400). The two proteins also co-elute in the CoFrac-MS dataset (Fig. 3*F*). YOP1 has been characterized as an ER-tubule-forming protein important for protein export in asexual parasites (75), and PF3D7_0912400 was previously identified as a putative interaction partner of ER chaperones (76). The AlphaFold 2 and AlphaFold 3 models of this complex yielded poor scores and did not satisfy the single interprotein cross-link, while the AF3x model satisfied the cross-links, but still with poor model confidence (Supplemental Table S3). Nevertheless, based on the cross-link and correlated co-elution profiles, YOP1- PF3D7_0912400 interaction represents a promising novel hypothetical complex.

As again displayed in Figure 2, box D, we also found cross-links between four components of the highly conserved Sec translocon (PF3D7_1438100, PF3D7_0821800, PF3D7_1346100, PF3D7_0204200), which transports proteins containing a signal peptide into the ER, where they are further processed and packaged into COPII vesicles for transport through the Golgi to the PPM (74). Related to this, Golgi protein 1 (PF3D7_1320000) and Golgi protein 2 (PF3D7_1123500) were cross-linked and co-elute in the CoFrac-MS dataset (Fig. 3*G*). Interaction between these two proteins in a Golgi apparatus protein complex, important for asexual replication, has been described previously (77). The detection of this complex in gametocytes points to a similar function during this stage of the life cycle. In line with this, we additionally observed a cross-link between the two transmembrane emp24 domain (TMED) proteins PF3D7_1314500 and PF3D7_1333300 (Fig. 2, box D). TMED proteins are a conserved protein family in eukaryotes and important regulators of protein transport between the ER and Golgi (78). Although neither protein has been described in detail in *P*. *falciparum*, PF3D7_1314500 has recently been investigated as a transmission-blocking vaccine candidate and was suggested as an early gametocyte-specific protein, indicating a function in gametocyte development (69, 79).

The highly conserved ER membrane complex (EMC) is an insertase in the ER membrane that, through its interaction with the SEC61 translocon, facilitates membrane protein assembly and folding (80, 81). In *P*. *falciparum*, it is putatively assembled from six subunits (EMC1/2/3/4/5/7 and 8) (16), and a loss of EMC3 function leads to a defect in gametocyte development with parasites displaying the absence of defined membrane structures (82). We identified a cross-linking network between the two EMC subunits, EMC1 (PF3D7_0811200), EMC3 (PF3D7_1360200) with the protein P22 (PF3D7_0208800) displayed in Figure 2, box D. HHpred (83) analysis of the P22 protein revealed similarities to the EMC10 subunit present in the *Saccharomyces* cerevisiae EMC complex (80) and indicates that P22 may be a homolog of EMC10, and form part of the *P*. *falciparum* EMC complex.

Another potentially high-value cross-linking subnetwork is composed of a protein with unknown function (PF3D7_0933300), the disulfide isomerase PfPDI-8 (PF3D7_0827900), a CLPTM1-domain containing protein (PF3D7_1137300), and the ER-resident calcium binding protein PfERC (PF3D7_1108600) (Fig. 2, box D). PfERC is involved in the processing and activation of proteases important for parasite egress from the iRBC and is a possible target of endoperoxides such as artemisinin (84, 85). It has also been shown to colocalize with PfPDI-8 (85), which is thought to play a crucial role in protein folding (86, 87). Although there is currently no functional data on PF3D7_1137300 and PF3D7_0933300, CLPTM1-domain proteins are known ER transmembrane proteins involved in lipid handling, protein trafficking, and glycosylphosphatidylinositol (GPI) anchor synthesis in the ER (88, 89). The observed cross-links between these proteins therefore point to their assembly in previously undescribed ER complexes potentially involved in protein processing and trafficking through the ER in gametocyte stages. These interactions are further supported by the observed co-elution of PF3D7_1137300 and PF3D7_0827900 in the CoFrac-MS dataset (Fig. 3*H*). An AF3x model of the PfPDI-8-PF3D7_1137300 complex satisfied 9 of 12 interprotein and all 14 intraprotein cross-links (Fig. 4*D*).

#### Proteasome, Mitochondria, and Miscellaneous

During gametocyte development, the parasites need to generate a large number of proteins to remodel the RBC as well as sustain the morphological changes during the development process. The regulation of protein turnover by the proteasome thus plays a crucial role during these life cycle stages, which is further emphasized by successful approaches to use the proteasome as a target for gametocidal drugs (90, 91). In addition, gametocytes also alter the size and morphology of the mitochondrion compared to the asexual parasite stage (65). This is caused by a shift to energy production through respiration and results in expanded mitochondria with cristae, which are absent in asexual stage parasites. Very recent data furthermore suggested the presence of multiple mitochondria in mature gametocytes (65). The proteasome, as well as the protein composition and molecular structure of the mitochondrion, have been described in detail in recent studies (16, 65). Accordingly, we identified many cross-links within the proteasomal or mitochondrial machinery, as well as several cross-links between proteins with unclear subcellular localization or function (Fig. 2, box E, Table1).

A notable example is cross-links between PF3D7_0617000 and PF3D7_0524700, which are the *P*. *falciparum* homologs of the translocase of outer membrane 40 (TOM40) and TOM20 (92). The interaction of these proteins is well described in other organisms, where they form parts of the conserved TOM complex responsible for protein transport across the outer mitochondrial membrane (93). The presence of this complex in *P*. *falciparum* has been proposed, but has lacked experimental evidence so far (92).

Another cross-linking network related to mitochondrial biology has been found between Stomatin-like protein PfSTOML (PF3D7_0318100) and the two prohibitins PfPHB1 and PfPHB2 (PF3D7_0829200 and PF3D7_1014700). All three proteins have been shown to localize to the mitochondria in gametocytes in *P*. *falciparum*, as well as *P*. *berghei*, where they play important roles during the formation of cristae and the maintenance of the mitochondrial integrity (94, 95, 96). Although an interaction between PfPBH1 and PfPBH2 has been described before, the interaction with PfSTOML has so far not been observed in the parasites. However, this interaction is described in HeLa cells, where the interaction of the PfSTOML homolog SLP-2 with PBH1 and PBH2 was shown to be important for stability of the prohibitins and the respiratory complex subunits in the inner mitochondrial membrane (95, 97). The observed cross-links between the three proteins thus suggest the presence of a similar interaction in *P*. *falciparum* gametocytes.

## Discussion

More than 20% of *P*. *falciparum* proteins still lack any functional annotation, and their roles in parasite biology remain unclear (98). Elucidating the function of these often-divergent proteins has the potential to identify promising targets for future intervention strategies. The systematic identification of protein–protein interaction networks at scale through proteomics provides a way to assign putative functions to unknown proteins by associating them with proteins of known function. So far, however, experimental studies of protein interactions on a proteome scale are very limited in *P*. *falciparum* and have only been conducted using a yeast-two hybrid screen or very recently native CoFrac-MS on asexual RBC stages (15, 99). Although informative, these methods have limitations with both methods often missing weak or transient interactions and generating false positives. Furthermore, they do not provide structural information on detected complexes. Gaps in our understanding of parasite protein complexes are particularly pronounced for the gametocyte stages, with proteomic studies and targeted interaction studies using affinity purification-MS or proximity-labeling approaches focused on the more accessible asexual stages (15, 100). Gametocyte-related studies have largely been restricted to comparative analyses between asexual and sexual stage proteomes or the identification of sex-specific proteins (100), while interaction data have come mainly from targeted co-immunoprecipitation experiments of a limited number of proteins. Only very recently has this been expanded by using native CoFrac-MS approaches to investigate protein–protein interactions in the overall gametocyte proteome and the mitochondria (16, 17).

Here, we applied an integrative approach to map membrane complexes in *P*. *falciparum* gametocytes. This strategy yielded a unique dataset that contains an extensive catalog of membrane proteins from the early gametocyte stages, providing an overview of the membrane-associated proteome. By combining CoFrac-MS with XL-MS and AlphaFold-based modeling, we assembled complementary evidence for multiple candidate complexes, capturing not only pairwise associations but also residue-residue level proximities and predicted interaction sites for a subset of these membrane proteins. Supplemental Table S4 summarizes the evidence supporting each candidate protein pair across XL-MS, CoFrac-MS, and AlphaFold-based modeling, and additionally indicates whether the pair is supported by XL-MS at 5% PPI-level FDR. Pairs lacking 5% PPI-level XL-MS support are presented as multievidence candidate associations rather than as XL-MS-only validated PPIs. Together, our data provides an extensive snapshot of protein–protein interactions in the membrane compartments of *P*. *falciparum* gametocytes, an area that has traditionally been neglected due to the inherent difficulties of investigating membrane proteins. The interactions span all gametocyte subcompartments and include newly identified complexes. Some of these are putatively involved in essential processes such as remodeling of the RBC cytoskeleton or transmembrane transport. The identification and our initial characterization of complexes provide a foundation for future studies on their biological functions.

Although single proteomics techniques carry a risk of false-positive interactions, the complementary support from XL-MS, CoFrac-MS, and AlphaFold modeling across different complexes strengthens confidence in the identified interactions. The structural models presented in this study are predictions and not experimentally determined complexes. Their reliability is nevertheless supported by several factors. Models selected for analysis consistently achieved high AlphaFold confidence scores (pLDDT, ipTM, pTM, or PAE around interfaces), which correlate well with deviations from experimentally solved structures (19, 20). Some models presented in Figure 4 exhibited low ipTM scores but obtained good pLDDT and PAE scores, and agreement between the three different AlphaFold versions. Many models also satisfied the majority of cross-linking restraints, and in several cases, they recapitulated known interactions or mapped accurately onto available structures. To minimize overinterpretation, only models with a good combined AlphaFold score and cross-link support were included in our main analysis, while less well-supported predictions are provided as a resource for future testing. This approach strengthens the interpretation of the proteomic data and offers structural hypotheses for complexes that remain experimentally inaccessible.

In this study, structural modeling and cofractionation data were not used to define binary acceptance criteria for protein–protein interactions, but rather to contextualize cross-linked protein pairs with complementary evidence. Defining quantitative thresholds would require calibrated cutoffs and a curated reference set of true and false complexes (101) with substantial overlap to the *P*. *falciparum* gametocyte proteome, which is currently not available. Moreover, commonly used AlphaFold interface score cutoffs show considerable overlap between true and false positives (102). We therefore report the interactions together with the associated cofractionation and modeling metrics and provide a consolidated summary table to enable informed assessment of individual cases (Supplemental Table S4).

CoFrac-MS combined with XL-MS represents a powerful strategy for untargeted mapping of protein–protein interactions and for inferring functions of poorly characterized proteins. In this study, we show that the approach can be effectively applied to *P*. *falciparum*, uncovering new protein–protein interactions, including those involving membrane proteins. Our findings suggest this strategy can be generalized to other parasite subcompartments and life-cycle stages, enabling faster and more comprehensive functional annotation of uncharacterized proteins in the *P*. *falciparum* proteome.

In summary, this study delivers the first large-scale, structurally informed map of membrane protein interactions in *P*. *falciparum* gametocytes. By combining cross-linking proteomics, cofractionation, and AlphaFold-based modeling, we establish a resource that expands the understanding of gametocyte membrane biology and offers a framework for exploring novel protein complexes with potential relevance to parasite development and transmission.

## Data Availability

The mass spectrometry proteomics data have been deposited to the ProteomeXchange Consortium via the PRIDE (103) partner repository with the dataset identifier PXD068680 for the CoFrac-MS dataset, and PXD067699 for the XL-MS datasets. Structural models are available on Zenodo at https://doi.org/10.5281/zenodo.19462728.

## Code Availability

Not applicable - only third-party software listed in the Methods section was used.

## Supplemental Data

This article contains supplemental data.

## Declaration of Generative AI and AI-Assisted Technologies

During the preparation of this work, the authors used ChatGPT and Grammarly to improve the style and grammar of the text. After using this tool or service, the authors reviewed and edited the content as needed and take full responsibility for the content of the publication.

## Conflict of Interests

The authors declare no competing interests.
