# Supplementary material for: Deciphering Membrane Protein Complexes in Plasmodium falciparum Gametocytes via Integrative Structural Systems Biology
Source: Mol Cell Proteomics. 2026 Apr 9;25(5):101567. doi: 10.1016/j.mcpro.2026.101567 (PMC13200099; doi:10.1016/j.mcpro.2026.101567)
Supplement: Supplemental Material 6 [file mmc6.pdf]

Supplementary Materials and figures

Supplementary Data Table 1 (Excel file). Crosslinking mass spectrometry data set. **A**, All identified crosslinks at 2% false discovery rate (FDR). **B**, Unique crosslinks retaining only the highest-scoring crosslink for each residue–residue pair. **C**, Expanded unique crosslinks where ambiguous protein assignments are resolved to all possible residue pair combinations (see Methods). **D**, Summary table listing inter-protein pairs and their crosslink counts. **E**, All crosslinked spectrum matches (CSMs) representing individual MS/MS identifications at 2% FDR. **F**, Protein-protein interactions (PPIs) identified at 5% FDR.

Supplementary Data Table 2 (Excel file). CoFrac-MS results

Supplementary Data Table 3 (Excel file). Scores and crosslink satisfaction rates of structural models built using AlphaFold. A, Models built using AlphaFold 2, **B**, Models built using AlphaFold 3. **C**, Models built using AF3x and all crosslinks.

Supplementary Data Table 4 (Excel file). A summary of all PPIs

Supplementary Data Table 5 (Excel file). Protein composition of complexes and correlations from Supp. Fig 5

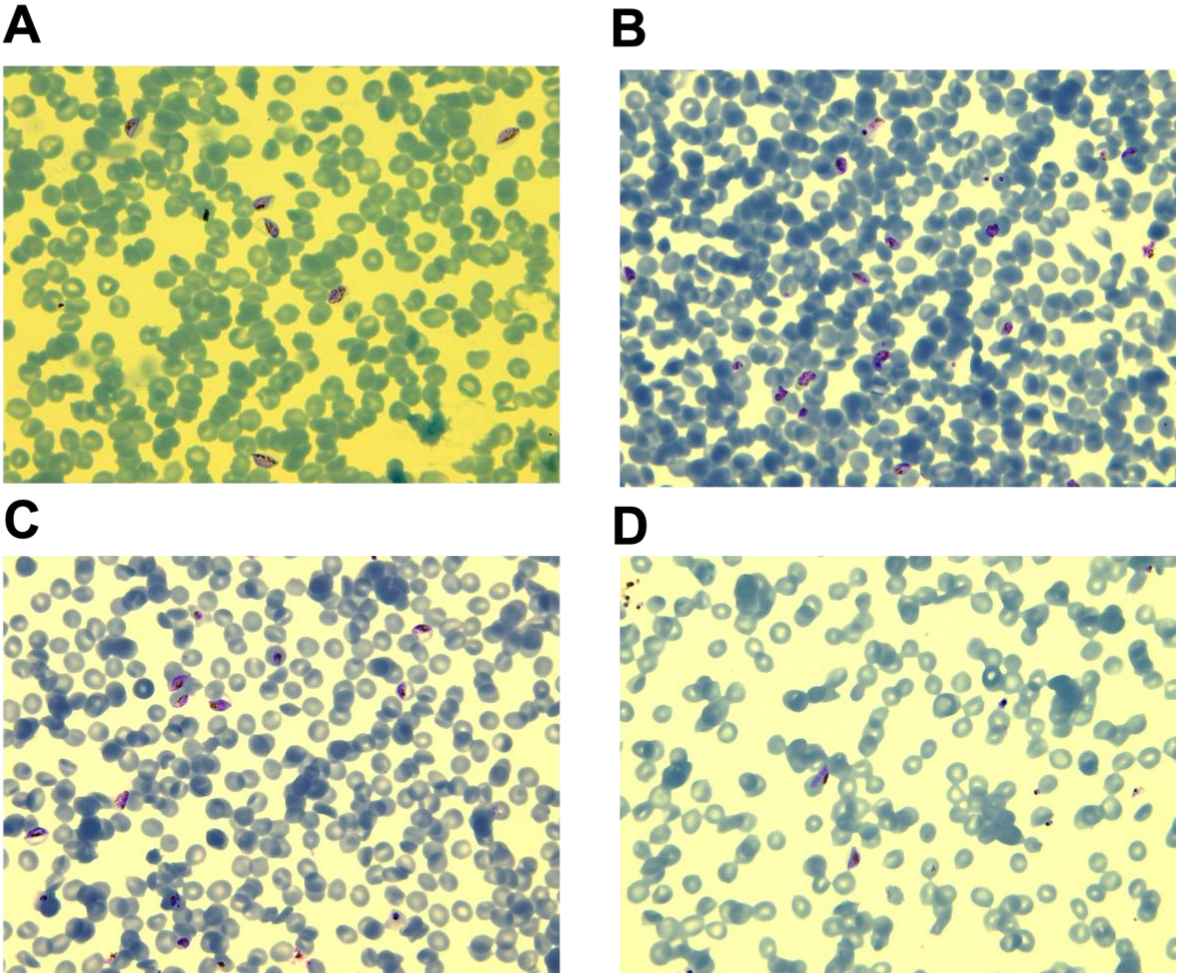

Supplementary figure 1: Smears from the cultures used in the various experiments. **(A)** shows the smear from the CoFrac-MS experiment, while **(B)**-**(D)** shows each of the batches that were combined for the XL-MS dataset. Smears illustrate the correct staging of the gametocytes at the time of sampling and the absence of asexually replicating parasites. Uninfected RBCs were removed and parasites released from the RBCs during the sampling by saponin treatment.

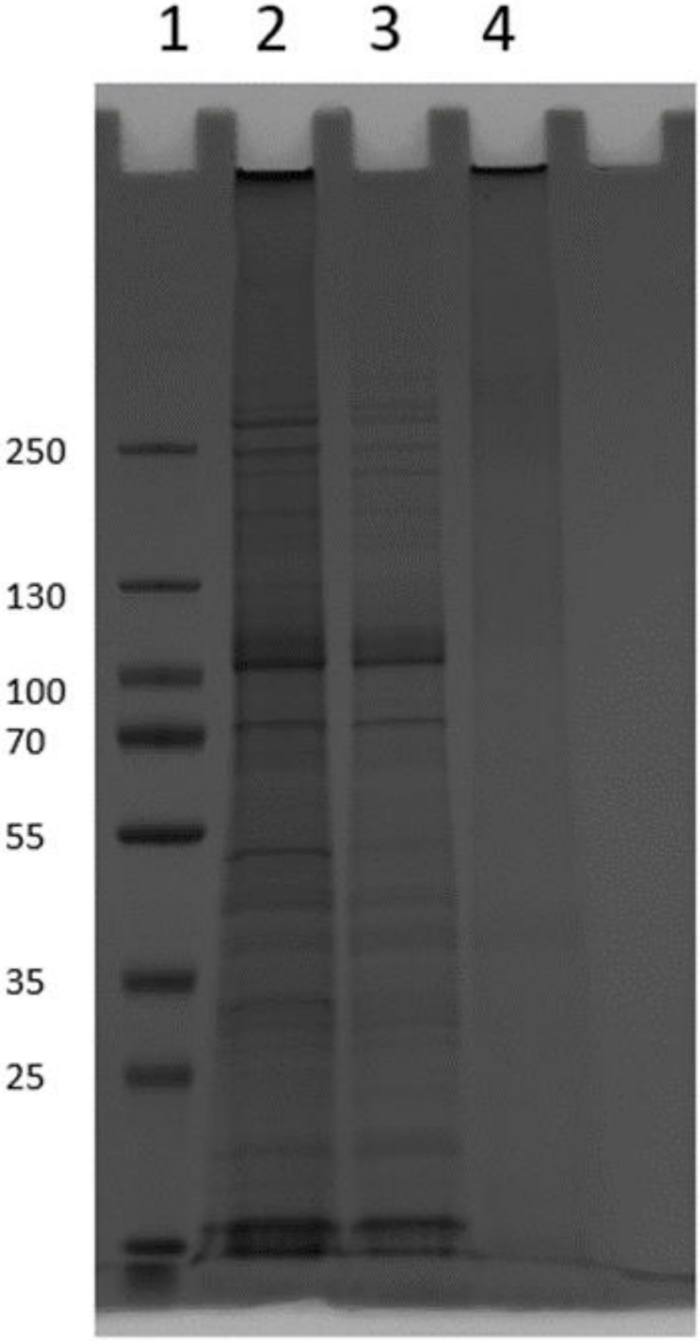

Supplementary figure 2: SDS-PAGE analysis of the membrane fractions before and after crosslinking with DSSO. Lane 1 is the ladder, Lane 2 shows the crude membranes, Lane 3 shows the membranes after solubilization and clarification, and Lane 4 shows the crosslinked sample.

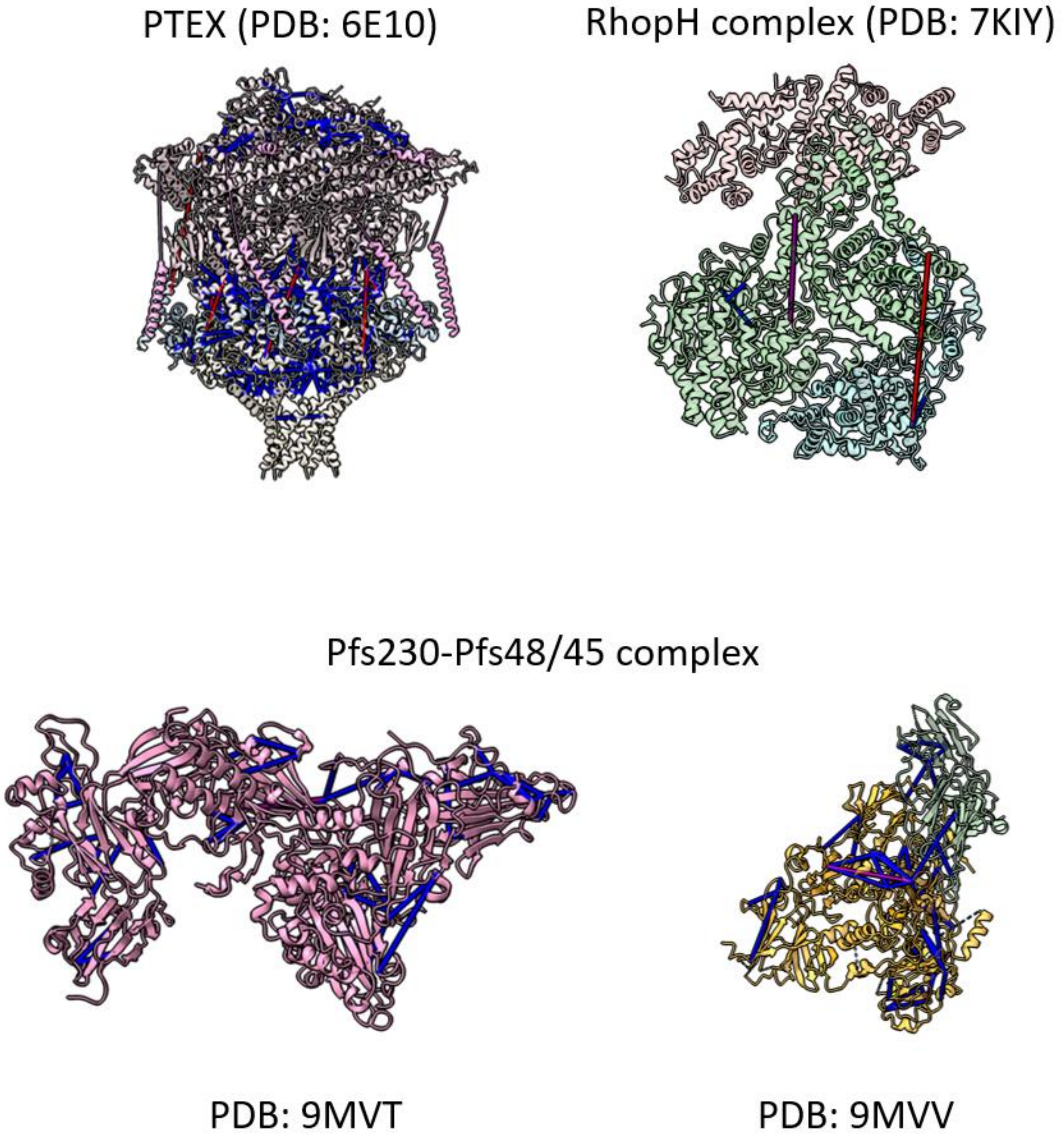

Supplementary figure 3: Gallery of cross-links mapped onto previously published structures. For PTEX (PDB 6E10), 35 of 37 cross-links are satisfied. For the RhopH complex (PDB 7KIY), 4 of 5 cross-links are satisfied. For the Pfs230–Pfs48/45 complex, 42 of 43 cross-links are satisfied in PDB 9MVT and 41 of 42 cross-links are satisfied in PDB 9MVV. The high fraction of satisfied cross-links supports the reliability of the datasets.

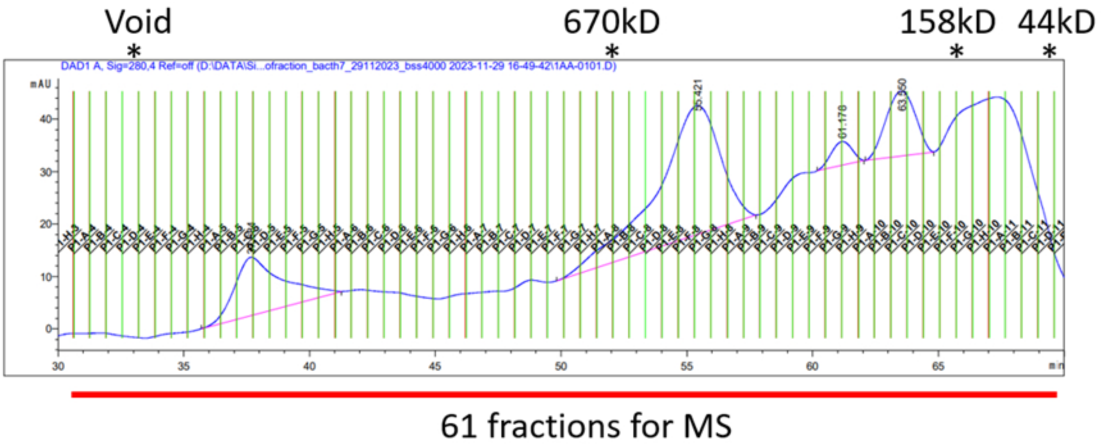

Supplementary figure 4: Chromatogram from the CoFrac-MS experiment.

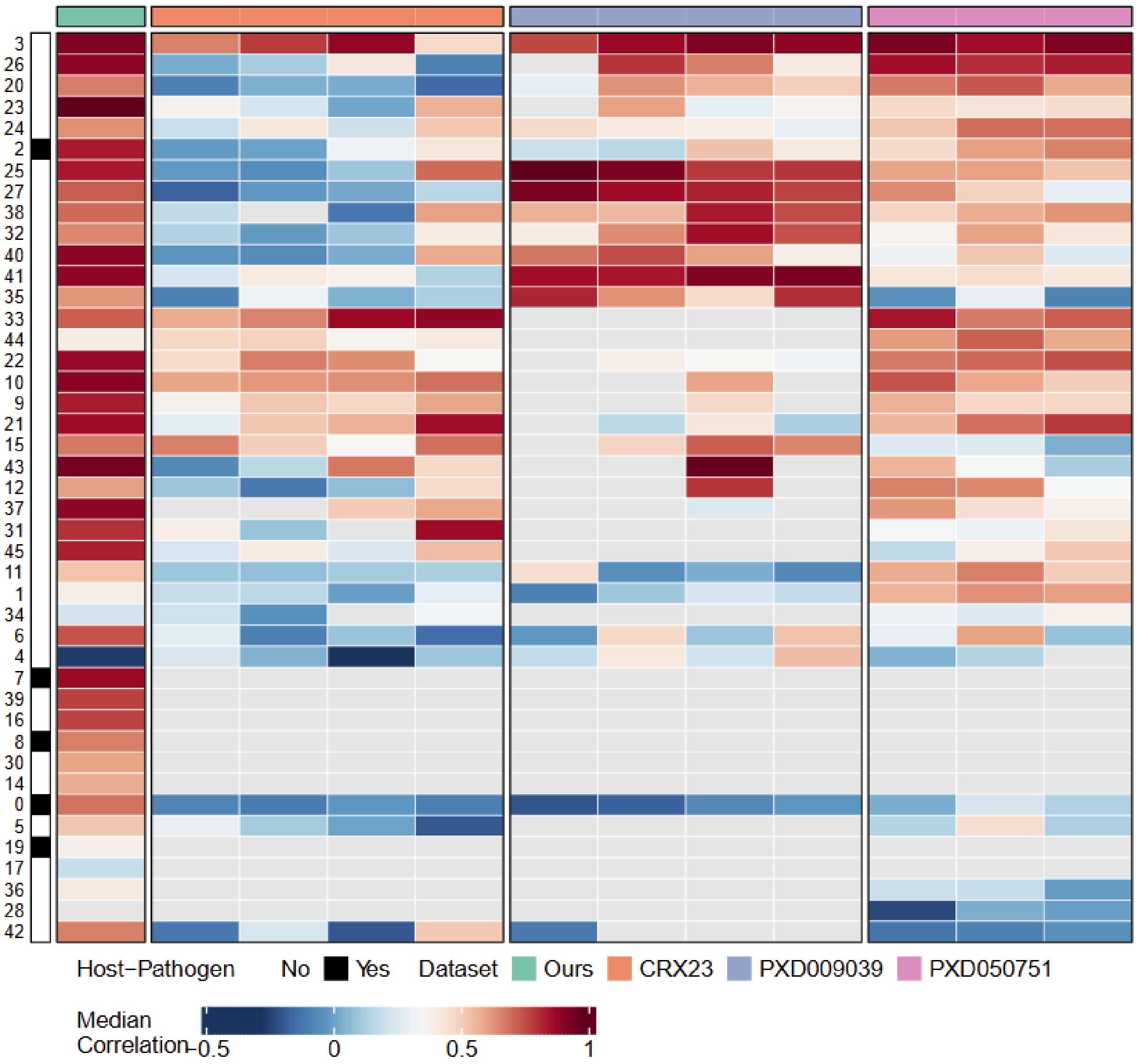

Supplementary figure 5: Correlative co-elution assessment of XL-MS-derived complexes across co-fractionation datasets. Shown is the median pairwise Spearman rank correlation of elution profiles for all crosslinked protein pairs within each inferred complex (rows), evaluated across our CoFrac-MS dataset and publicly available CoFrac-MS datasets from the literature (columns). Complex identifiers correspond to connected components derived from the XL-MS interaction network; the protein composition of each complex is listed in Supp. Data Table 5. Because of differences in parasite stages, experimental workflows, and data processing between studies, correlations are intended as qualitative indicators of co-elution consistency rather than quantitative measures of interaction confidence. (CRX23: data from late-stage *P. falciparum* gametocytes; PXD009039: data from late-stage *P. falciparum* gametocytes; PXD050751 data from *P. falciparum* schizonts)

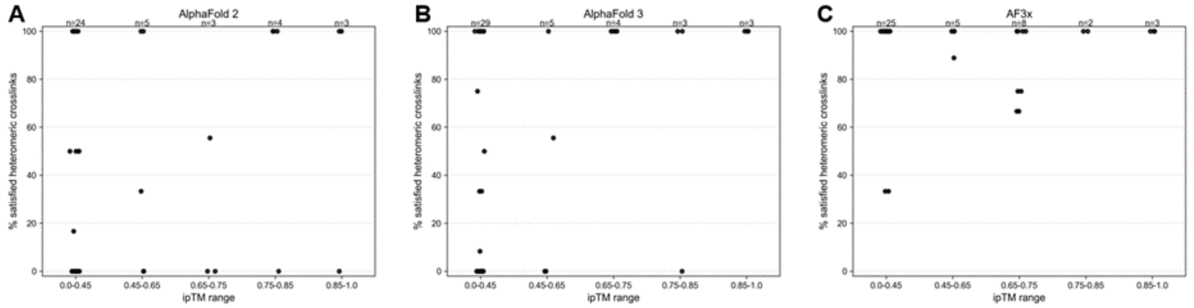

Supplementary figure 6: Overview of the modeling results and agreement of AlphaFold 2 **(A)**, AlphaFold 3 **(B)**, and AF3x **(C)** with XL-MS for dimeric complexes. Each panel shows the percentage of satisfied heteromeric crosslinks per prediction, binned by ipTM (0–0.45, 0.45–0.65, 0.65–0.75, 0.75–0.85, 0.85–1.0). Dots are individual predictions; horizontal jitter is added to reduce overlap. Numbers above bins indicate n.
